# Nitidine chloride suppresses polo-like kinase 1 via MYCN-associated transcriptional regulation in colorectal cancer: a multi-omics and spatial transcriptomics study

**DOI:** 10.3389/fonc.2026.1824796

**Published:** 2026-05-12

**Authors:** Xiao-Jue Huang, Liu-Hui Mo, Ke-Jun Wu, Rong-Quan He, Hui Li, Gang Chen, Li-Min Liu

**Affiliations:** 1 Department of Toxicology, College of Pharmacy, Guangxi Medical University, Nanning, Guangxi, China; 2 Department of Pharmacy, The People’s Hospital of Guangxi Zhuang Autonomous Region & Guangxi Academy of Medical Sciences, Nanning, Guangxi, China; 3 Department of Pharmacy, The Rui Kang Hospital Affiliated to Guangxi University of Chinese Medicine, Nanning, Guangxi, China; 4 Department of Pathology, The First Affiliated Hospital of Guangxi Medical University, Guangxi Medical University, Nanning, Guangxi, China; 5 Department of Medical Oncology, The First Affiliated Hospital of Guangxi Medical University, Nanning, Guangxi, China; 6 Department of Colorectal and Anal Surgery, The First Affiliated Hospital of Guangxi Medical University, Nanning, Guangxi, China

**Keywords:** colorectal cancer, molecular dynamics simulation, multi-omics, MYCN, nitidine chloride, polo-like kinase 1, spatial transcriptomics

## Abstract

**Background:**

Colorectal cancer (CRC) remains a major global health burden. While nitidine chloride (NC) exhibits anti-tumor effects, its molecular targets in CRC are still largely unknown.

**Methods:**

To delineate the role of Polo-like kinase 1 (PLK1) in CRC, we combined RNA sequencing with comprehensive multi-omics integration across 3,513 specimens (2,256 tumor and 1,257 non-tumor). Single-cell and spatial transcriptomic approaches were employed to map PLK1 expression heterogeneity and its precise localization within tumor architecture. Direct interaction between NC and PLK1 was evaluated through 100-ns GROMACS molecular dynamics simulations of the PLK1 structure retrieved from the Protein Data Bank structures and MM-PBSA binding free energy analysis. Upstream regulation by MYCN was probed via public ChIP-seq datasets, E-box motif scanning, and AlphaFold-Multimer docking. Functional validation included RT-qPCR quantification of PLK1 mRNA in NC-treated HCT116 cells and dose-dependent IHC analysis of PLK1 protein in HCT116 xenograft tumors following NC or 5-FU administration.

**Results:**

PLK1 was identified as a prominent NC-responsive gene and was consistently overexpressed in CRC across multiple datasets. Enrichment analyses indicated that PLK1-associated genes were mainly involved in cell cycle-related pathways. Molecular dynamics simulations supported a stable interaction pattern between NC and PLK1. Single-cell and spatial transcriptomic analyses showed that PLK1 expression was enriched in malignant epithelial cells and proliferative tumor regions and displayed marked intratumoral heterogeneity. NC treatment significantly reduced PLK1 expression at both the mRNA and protein levels. In addition, MYCN was concurrently downregulated after NC treatment, and integrated public-cohort analyses showed that MYCN was positively correlated with PLK1 expression in CRC. Combined with genome browser visualization, motif analysis, and structural modeling, these findings suggest a potential association between MYCN and PLK1 regulatory activity.

**Conclusions:**

This study identifies PLK1 as an important candidate target of NC in CRC and demonstrates that NC suppresses PLK1 expression at both the transcript and protein levels. Integrative analyses further indicate that the MYCN–PLK1 axis may represent a potential regulatory component associated with the anti-CRC effects of NC. Nitidine chloride; Polo-like kinase 1; MYCN; Molecular dynamics simulation; Multi-omics; Spatial transcriptomics; Colorectal cancer.

## Introduction

Colorectal cancer (CRC) ranks third among global malignancies and continues to drive rising incidence and mortality (1, 2). Long-term survival remains poor in advanced cases (3), and standard chemotherapeutics like 5-FU are limited by toxicity, highlighting the urgent demand for novel targets and better therapies.

Natural compounds, valued for their diversity and low toxicity, have demonstrated significant anti-tumor potential. Nitidine chloride (NC) induces apoptosis, inhibits proliferation, and arrests the cell cycle, showing efficacy in liver cancer (4–6). Yet its mechanisms in colorectal cancer are still unclear.

Polo-like kinase 1 (PLK1) serves as a master regulator of mitotic progression (7, 8), orchestrating critical events such as centrosome maturation, spindle formation, chromosome alignment, and cytokinesis. Dysregulated PLK1 expression contributes to oncogenesis and tumor progression in multiple malignancies (9, 10). In colorectal cancer, PLK1 overexpression promotes aggressive proliferation and is strongly associated with poor clinical prognosis. Consequently, PLK1 has been recognized as a compelling druggable target, and PLK1 inhibitors have yielded encouraging results in early-phase clinical trials (11, 12).

To clarify the mechanism of action of NC against colorectal cancer, this study systematically investigated the inhibitory effect of compound NC on CRC HCT116 cells and its molecular mechanism through *in vitro* cell experiments, *in vivo* animal models, MDS, and multi-omics analysis and other technical means.

## Methods

### RNA sequencing and differential gene expression analysis

HCT116 cells in the logarithmic growth phase were divided into control (0.1% DMSO) and NC treatment (6 µmol/L) groups, with three independent biological replicates per group. After seeding into six-well plates and reaching ~60% confluence, cells were treated for 48 h at 37 °C in a 5% CO_2_ incubator. Total RNA was extracted using the AxyPrep™ Multisource Total RNA Miniprep Kit. RNA samples were sent for commercial whole-genome mRNA sequencing. Library construction included mRNA purification, fragmentation, reverse transcription, adapter ligation, and PCR amplification. Raw reads underwent quality control with FastQC, trimming with Trimmomatic, and alignment to the human reference genome using HISAT2. Gene-level counts were generated with featureCounts. Differential expression analysis was performed using DESeq2 (v1.34.0) with the Wald test, and p-values were adjusted using the Benjamini-Hochberg method. Genes with padj < 0.05 and |log_2_FC| > 1 were considered significantly differentially expressed. Downregulated genes were subjected to GO and KEGG enrichment analysis.

### Molecular dynamics simulation methods

In biology and drug design, MD simulation has emerged as a powerful tool for studying interactions between biomacromolecules (e.g., proteins and nucleic acids) and small molecules (e.g., drug candidates). Using advanced software such as GROMACS, researchers can simulate the dynamic behavior of molecules in environments approximating real biological conditions, thereby deepening understanding of their functions and interaction mechanisms. In this study, the three-dimensional structure of PLK1 was retrieved from the RCSB Protein Data Bank database (PDB ID: 2RKU), and the small molecule NC was obtained from PubChem (CID: 4501). Comprehensive 100 ns MD simulations were then performed on the protein–ligand complex obtained from molecular docking using GROMACS 2024.2, the industry-leading MD simulation package.

Small-molecule preprocessing is a critical step to ensure simulation accuracy and reliability. The ligand NC was prepared with sobtop to apply the General Amber Force Field parameters and assign atomic charges, while the protein system was parameterized using the AMBER14SB force field combined with the TIP3P water model (13) The system was neutralized by adding an appropriate number of Na^+^ ions to achieve electroneutrality. These operations optimized molecular geometry and ensured accurate charge distribution, providing a solid foundation for subsequent simulations. Simulation conditions closely mimicked physiological environments: a constant temperature of 300 K and pressure of 1 bar. Long-range electrostatic interactions were computed via the particle mesh Ewald (PME) method with a 1.2 nm cutoff, and non-bonded interactions were truncated at 10 Å (14). Hydrogen-containing bonds were constrained using the LINCS algorithm with a 2 fs time step (15). System temperature was maintained with the V-rescale thermostat, and pressure was controlled at 1 bar using the Berendsen barostat.

Prior to production runs, the system underwent energy minimization via the steepest descent algorithm to eliminate conformational strain and unfavorable geometries. Equilibration was then performed in the isothermal-isochoric (NVT) and isothermal-isobaric (NPT) ensembles for 500 ps each (250,000 steps, coupling constant 0.1 ps) to stabilize temperature and pressure. Finally, unrestrained MD production simulation was conducted for 50,000,000 steps (2 fs timestep), totaling 100 ns, to fully capture the system’s dynamic behavior.

Trajectory analysis was performed using VMD and PyMOL (16, 17). Structural stability and dynamics were evaluated via root-mean-square deviation (RMSD), radius of gyration (Rg), and root-mean-square fluctuation (RMSF) (18), supplemented by principal component analysis (PCA) (7) and free energy landscape (FEL) analysis. Binding free energy was calculated using the Molecular Mechanics Poisson–Boltzmann Surface Area (MM-PBSA) method via the g_mmpbsa tool (16, 17) to quantify the binding affinity and thermodynamic stability between NC and PLK1. Additional metrics—including buried solvent-accessible surface area (SASA), center-of-mass evolution, hydrogen bond numbers, electrostatic interaction energies, and van der Waals interaction energies—were calculated to comprehensively characterize protein–ligand interactions (19–21).

The RMSD is a key metric for quantifying conformational stability and similarity between structures, with lower RMSD values indicating smaller deviations and higher structural similarity (unit: Å). The RMSF measures the positional fluctuation amplitude of individual amino acid residues over the simulation period, reflecting local flexibility influenced by both intrinsic polypeptide chain properties and environmental factors. The Rg quantifies the compactness of the protein structure as the root-mean-square distance of atoms from the molecular center of mass; more stable structures typically exhibit smaller Rg values. SASA represents the surface area of the macromolecule exposed to solvent and is largely determined by surface hydrophilicity. Smaller SASA values indicate tighter folding and reduced solvent exposure, thereby enhancing thermal stability and functional integrity.

### ScRNA-seq analysis of PLK1 expression in CRC

Publicly available scRNA-seq data for CRC were downloaded from the GEO under accession numbers GSM6061702 and GSM6061703. Raw count matrices were processed using the Seurat R package (v5.0). Low-quality cells were filtered by removing genes detected in fewer than 3 cells and cells expressing fewer than 50 genes. Cells with more than 200 genes detected and mitochondrial gene content below 25% were retained. After normalization and scaling, the top 3000 highly variable genes were identified and used for principal component analysis (PCA), followed by UMAP dimensionality reduction and graph-based clustering. Cell types were annotated based on canonical marker genes.

To distinguish malignant epithelial cells from normal epithelial cells, inferCNV analysis was performed. Malignant colonocyte-like, epithelial, stem-like, goblet-like, and proliferative clusters were selected together with B cells (as the normal reference group). Gene order files were generated using the AnnoProbe package, and the inferCNV object was created with raw counts, cell annotations, and gene position information. The analysis was run in subclusters mode with cutoff = 0.1, denoise = TRUE, and HMM = FALSE. A median filter was subsequently applied to smooth the CNV profile. CNV scores were calculated by thresholding expression deviations relative to the B-cell reference (mean ± 2 SD), assigning scores of 0 (normal), 1 (single-copy gain/loss), or 2 (multi-copy gain/loss). The total CNV score per cell was written back to the Seurat object for downstream analysis. Malignant cells were defined as those with CNV scores above the median, and high-CNV malignant subclusters were extracted for further sub-clustering and visualization.

### mRNA level expression of PLK1 in CRC

Publicly available mRNA expression data for PLK1 in CRC versus non-CRC tissues were retrieved from GEO, ICGC, GTEx, SRA, TCGA, PubMed, and ArrayExpress using the search terms “colorectal cancer”, “colorectal carcinoma”, and “CRC”. Only datasets meeting the following criteria were included: (1) human primary CRC tissues, (2) paired tumor and normal colorectal samples, and (3) minimum of 3 samples per group.Datasets lacking PLK1 expression values or containing metastatic/recurrent samples were excluded. Before merging, a total of 28 independent datasets were retrieved (corresponding to 18 GEO platforms). To reduce technical heterogeneity while preserving biological variation, data from identical GEO platforms were merged as follows: GPL570 (Affymetrix Human Genome U133 Plus 2.0 Array) containing GSE39582, GSE17536, GSE41258, GSE23878, GSE103479, GSE110225, GSE87211, GSE72970, GSE87216, GSE113513, GSE117606, GSE122182; GPL96 (Affymetrix Human Genome U133A Array) containing GSE14333, GSE17538, GSE35834, GSE18088, GSE18105; GPL13158 (Affymetrix Human Gene 1.0 ST Array) containing GSE44076, GSE44861, GSE50710, GSE73360; GPL10558 (Illumina HumanHT-12 V4.0 Expression BeadChip) containing GSE49355, GSE21510, GSE10714, GSE8671; and GPL21290 (Illumina HiSeq 2500 RNA-seq) containing GSE50760, GSE79973, GSE101479, GSE116182.

These platforms were then normalized by Log2(x + 1) and batch-corrected with the limma and sva packages. The meta package (version 4.18-2) was used to compute standardized mean differences (SMD). Given the expected clinical and technical heterogeneity across platforms and cohorts, a random-effects model was pre-specified for all meta-analyses. Between-study variance (τ²) and I² statistics were calculated to quantify heterogeneity. For diagnostic performance evaluation, expression values were dichotomized at the median within each dataset to generate sensitivity and specificity pairs, and summary receiver operating characteristic (SROC) curves were constructed using the Moses-Littenberg method. The threshold for dichotomization was dataset-specific (median expression value).

### Protein level expression of PLK1 in CRC

PLK1 protein expression in CRC was validated by immunohistochemistry (IHC) using data from the Human Protein Atlas (HPA, antibody HPA053229). Additionally, quantitative proteomic data from the Proteomic Data Commons (PDC) were analyzed, comparing 40 normal and 42 CRC samples.

### Spatial transcriptome analysis of PLK1 expression

Spatial transcriptomics was employed to map the spatial distribution of PLK1 within tumor tissue. The public 10x Genomics Visium dataset VISDS000771 was processed with the SpaCET R package. A spatial data object was created with a minimum gene expression threshold of 1 to suppress noise. Quality control was performed by evaluating UMI counts per spot, number of detected genes (nFeature), H&E images, and spatial coordinates. After preprocessing, spatial expression maps were generated for PLK1 and the proliferation marker MKI67 to identify proliferative hotspots. Cell-type deconvolution was conducted to infer the spatial abundance of major immune populations (CD4+ naïve T cells, follicular B cells, cDC2, macrophages, NK cells, cytotoxic T cells, exhausted T cells, and proliferating T cells).

To rigorously quantify spatial autocorrelation, univariate Moran’s I statistics were computed for PLK1 expression and for a composite immune score (sum of the proportions of CD8+ T cells, CD4+ T cells, B cells, NK cells, and macrophages) using the spdep package with k-nearest neighbor spatial weights. Bivariate spatial autocorrelation between PLK1 expression and total immune infiltration was additionally assessed using Lee’s L statistic. Statistical significance was determined by permutation tests. To control for potential technical bias arising from variation in sequencing depth, log-transformed UMI counts were regressed out from both variables prior to recomputing Moran’s I and Lee’s L on the residuals.

Robustness was assessed by integrating eight deconvolution algorithms (CIBERSORT, CIBERSORT_ABS, EPIC, ESTIMATE, MCPcounter, quanTlseq, TIMER, and xCell) with correlation analysis.

### Effect of PLK1 expression and CRISPR gene knockout on CRC cell lines

The Dependency Map (DepMap) database was queried to assess PLK1 expression across CRC cell lines and the impact of CRISPR-mediated gene knockout on cell viability. DepMap integrates gene expression, mutation, and CRISPR screening data. Knockout screens were analyzed using the CERES algorithm to generate dependency scores. Negative scores indicate that PLK1 is essential for cell proliferation (knockout inhibits growth), whereas positive scores suggest a growth-suppressive role.

### RNA extraction and RT-qPCR

HCT116 cells were treated with NC (6 μmol/L) or 0.1% DMSO vehicle for 48 h (three biological replicates per group). Total RNA was extracted and evaluated for concentration and purity using a NanoDrop 2000 spectrophotometer, with GAPDH as the internal reference gene. Reverse transcription was performed using the PrimeScript RT Reagent Kit with gDNA Eraser (TaKaRa, RR047A). Quantitative PCR was carried out with PowerUp™ SYBR™ Green Master Mix following the manufacturer’s protocol. PLK1 primer sequences were: forward 5′-CTTTTTCGAGGACAACGACTTC-3′, reverse 5′-GATGAATAACTCGGTTTCGGTG-3′. Each experiment was independently repeated three times, and relative mRNA expression was calculated by the 2^(−ΔΔCT) method.

### Immunohistochemical staining

Immunohistochemical (IHC) staining was performed to assess PLK1 protein levels in tumor tissues from the HCT116 CRC nude mouse xenograft model (control, low-/medium-/high-dose NC, and 5-FU groups). Tissues were fixed, paraffin-embedded, sectioned, and dewaxed using standard protocols. Endogenous peroxidase and nonspecific binding were blocked, followed by incubation with rabbit anti-PLK1 polyclonal antibody (1:50 dilution; ProteinTech Group, Wuhan, China) and visualization with 3,3′-diaminobenzidine (DAB). Images were acquired on a Leica DM18 microscope at ×200 magnification. All procedures were conducted in triplicate under double-blind conditions. PLK1 expression was quantified by average optical density (OD) of positive staining using ImageJ software.

### MYCN binding to the PLK1 promoter and in silico modeling of MYCN–PLK1 regulatory interaction

Publicly available MYCN ChIP-seq datasets were analyzed to examine MYCN occupancy at the PLK1 genomic locus. Genome browser visualization was used to assess MYCN binding signals in relation to promoter regions and active chromatin marks. To identify potential MYCN binding sites, the promoter region of PLK1 was defined as the upstream sequence surrounding the transcription start site and scanned for canonical E-box motifs using the JASPAR database and motif enrichment tools.

To explore the structural basis of MYCN-mediated regulation, the MYCN–MAX heterodimer was modeled using AlphaFold-Multimer, reflecting the functional DNA-binding configuration of MYCN. The predicted protein complex was subsequently used for protein–DNA docking with the identified PLK1 promoter motif sequence to generate a putative interaction model. Structural confidence was evaluated based on predicted local distance difference test (pLDDT), inter-protein predicted TM-score (ipTM), and predicted aligned error (PAE) metrics. All structural visualization and analyses were performed using PyMOL.

### Correlation analysis between PLK1 and MYCN

To evaluate the association between PLK1 and MYCN expression, Pearson correlation analysis was performed between the two genes in each of the 14 publicly available CRC transcriptomic datasets. For each cohort, the correlation coefficient and raw p-value were calculated. To account for multiple testing, p-values were adjusted using the Benjamini-Hochberg false discovery rate (FDR) method across the 14 independent tests. Correlations with FDR < 0.05 were considered statistically significant.

### Statistical analysis

Differences in PLK1 expression between groups were assessed using the Wilcoxon rank-sum test, with statistical significance set at P < 0.05. Given the expected clinical and technical heterogeneity across different platforms and cohorts, a random-effects model was pre-specified for all meta-analyses regardless of the Cochran’s Q test result. Between-study variance (τ²) and I² statistics were calculated to quantify heterogeneity. For diagnostic performance evaluation, gene expression values were dichotomized at the dataset-specific median to generate sensitivity and specificity pairs for each cohort. Summary receiver operating characteristic (SROC) curves were then constructed using the Moses-Littenberg method in STATA 18.0. The area under the curve (AUC) was calculated to evaluate the ability of PLK1 expression to discriminate tumor from normal samples within each cohort. For SROC analysis, gene expression values were dichotomized at the dataset-specific median to generate sensitivity and specificity pairs. Publication bias was examined by Begg’s test, where P > 0.05 indicated no significant bias.

## Results

### PLK1 is a potential target of NC in HCT116 cells

**Figure 1:**
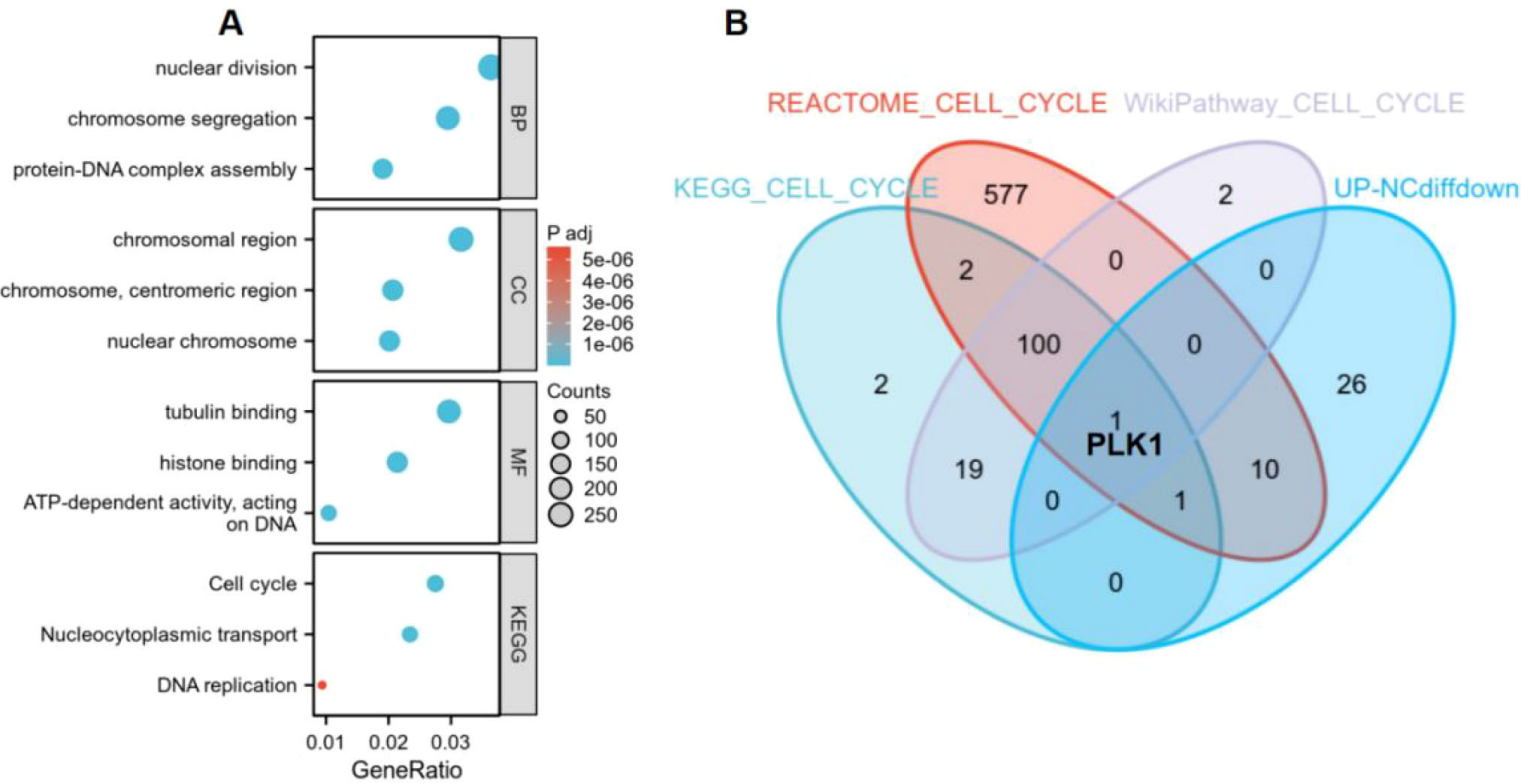
Functional enrichment analysis of genes downregulated by NC. **(A)** GO and KEGG enrichment analysis. **(B)** Venn diagram of cell cycle pathways. NC, Nitidine Chloride; GO, Gene Ontology; KEGG, Kyoto Encyclopedia of Genes and Genomes.

Downregulated genes after NC treatment were enriched in cell cycle processes (nuclear division, chromosome segregation, protein-DNA assembly). Cell cycle pathway was significantly enriched (P < 0.001) (**Figure 1A**). PLK1 was identified as key gene in KEGG, Reactome, and WikiPathway cell cycle pathways downregulated by NC (**Figure 1B**).

### NC–PLK1 interaction and dynamic stability analysis

**Figure 2:**
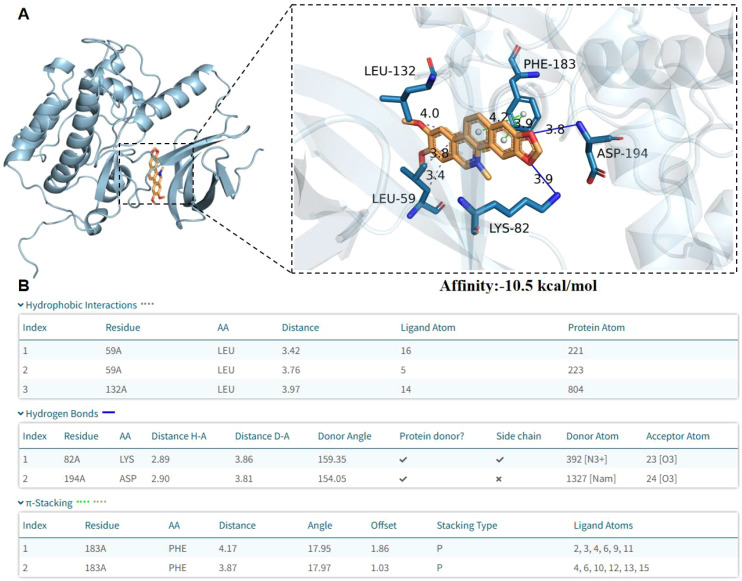
Molecular dynamics results of NC and PLK1. **(A)** Three-dimensional representation of the protein–ligand complex. The overall protein structure is shown in cartoon format (left), with the ligand depicted as sticks within the binding pocket. The right panel shows an enlarged view of the binding site, highlighting the spatial arrangement of the ligand and surrounding amino acid residues (e.g., LEU-59, LEU-132, LYS-82, PHE-183, and ASP-194), along with the distances of their interactions. **(B)** Summary of protein–ligand interaction analysis. The table presents hydrophobic interactions, hydrogen bonds, and π-stacking interactions, including details such as interacting residues, atom indices, interaction distances, angles, and related geometric parameters.

Molecular docking results showed that the binding affinity of NC to PLK1 reached -10.5 kcal/mol, indicating strong binding potential between the two (**Figure 2**). NC was embedded in the active pocket of PLK1 and formed multiple stable interactions with surrounding residues (**Figure 2A**). Key residues (LEU-59, LEU-132, LYS-82, PHE-183, and ASP-194) displayed clear spatial arrangements and interaction distances with NC (**Figure 2A**, enlarged view), where NC formed three stable hydrophobic contacts with LEU-59 (distances 3.42 Å and 3.76 Å) and LEU-132 (distance 3.97 Å) (**Figure 2B**, Hydrophobic Interactions). In addition, NC established two H-bonds with LYS-82 (H-A distance 2.89 Å, D-A distance 3.86 Å, donor angle 159.35°) and ASP-194 (H-A distance 2.90 Å, D-A distance 3.81 Å, donor angle 154.05°), with the LYS-82 side chain participating in H-bond formation (**Figure 2B**, Hydrogen Bonds). Furthermore, NC formed two parallel π-stacking interactions (P-type) with PHE-183 at distances of 4.17 Å and 3.87 Å, angles of approximately 18°, and offsets of 1.86 and 1.03, respectively (**Figure 2B**, π-Stacking).

**Figure 3:**
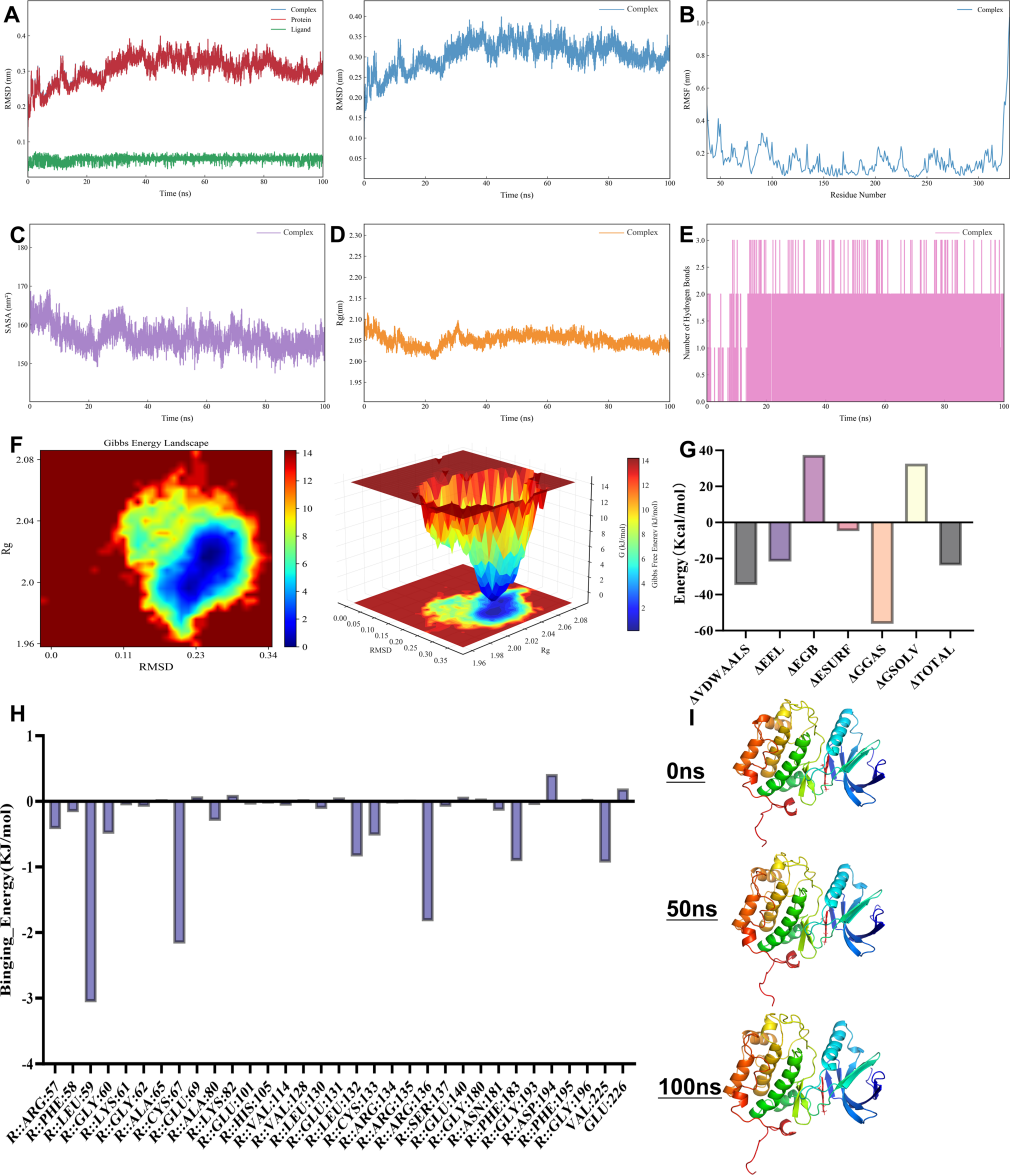
Molecular dynamics results of NC and PLK1. **(A)** Root mean square deviation (RMSD) of the complex, protein, and ligand as a function of simulation time. **(B)** Root mean square fluctuation (RMSF) of protein residues. **(C)** Solvent-accessible surface area (SASA) of the complex over time. **(D)** Radius of gyration (Rg) of the complex during the simulation. **(E)** Number of hydrogen bonds formed between the protein and ligand over time. **(F)** Gibbs free energy landscape as a function of RMSD and Rg, presented in both 2D contour and 3D surface plots. **(G)** Binding free energy components, including van der Waals, electrostatic, polar solvation, nonpolar solvation, and total energy contributions. **(H)** Per-residue binding energy contribution of amino acids to the ligand. **(I)** Representative structures of the protein–ligand complex at 0 ns, 50 ns, and 100 ns during the simulation. NC, Nitidine Chloride; PLK1, polo-like kinase 1; PCA, principal component analysis; RMSD, root mean square deviation; Rg, radius of gyration; RMSF, root mean square fluctuation.

To further validate the dynamic stability and binding mechanism of the NC–PLK1 complex under physiological conditions, 100 ns MD simulations were performed. RMSD curves of the complex, protein, and ligand rapidly equilibrated in the early stage of the simulation and then remained at low levels with a tendency to stabilize, indicating that the overall conformation of the system exhibited no significant drift and the structure remained highly stable throughout the simulation (**Figure 3A**). RMSF analysis revealed that most residues of PLK1 showed small fluctuation amplitudes, especially the key residues in the ligand-binding pocket (LEU-59, LYS-82, LEU-132, PHE-183, and ASP-194), whose RMSF values stayed low, confirming that these residues maintained good rigidity and sustained stable interactions with NC (**Figure 3B**). SASA curves fluctuated minimally and remained stable throughout the simulation, suggesting tight folding of the complex with no significant change in solvent exposure (**Figure 3C**). Similarly, the Rg curve stayed steady, indicating that the overall protein structure remained compact without obvious extension or collapse (**Figure 3D**). The time-dependent curve of H-bond numbers between protein and ligand showed an average of 2–3 H-bonds maintained during the simulation, highly consistent with the molecular docking results and further confirming the persistence of H-bonds (**Figure 3E**). PCA and FEL based on RMSD and Rg presented a single stable low-energy conformational basin, demonstrating that the complex mainly existed in one dominant conformation with favorable thermodynamic stability (**Figure 3F**). MM-PBSA binding free energy decomposition indicated that van der Waals forces and electrostatic interactions were the primary driving forces, with a negative total binding free energy confirming the spontaneous binding of NC to PLK1 (**Figure 3G**). Per-residue energy contribution analysis further revealed that key residues LEU-59, LEU-132, LYS-82, PHE-183, and ASP-194 contributed most to the total binding energy, fully consistent with the interaction analysis from molecular docking (**Figure 3H**). Representative conformations extracted from the trajectory at 0 ns, 50 ns, and 100 ns showed that NC remained stably positioned in the PLK1 active pocket, with no significant changes in the spatial positions or interaction patterns of key residues (**Figure 3I**). Collectively, these MD simulation results demonstrate that the NC–PLK1 complex exhibits excellent structural stability and dynamic binding characteristics over 100 ns, providing robust computational evidence for the potential application of NC as a PLK1 inhibitor.

### Multi-omics analysis confirms high expression of PLK1 in CRC

**Figure 4:**
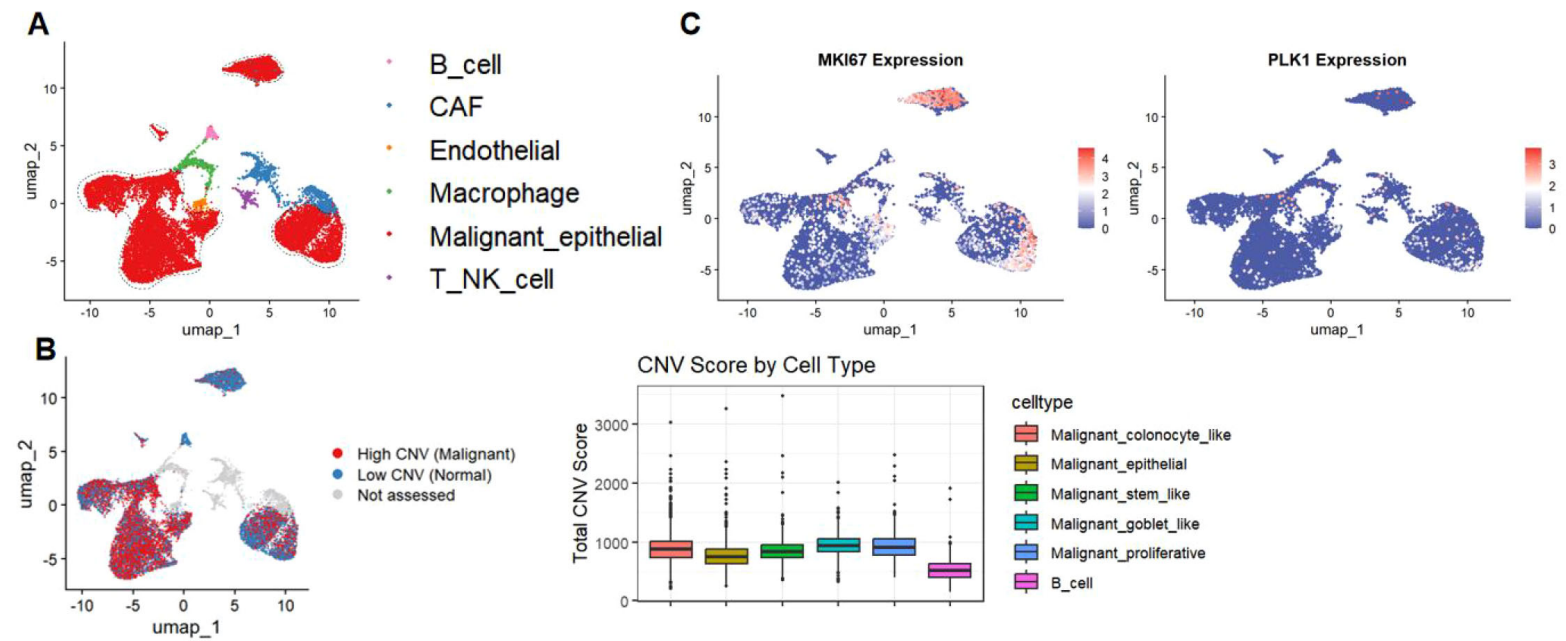
Single-cell identification of malignant cell populations and proliferation-associated gene expression in colorectal cancer (CRC). **(A)** UMAP visualization showing the major cell populations in CRC samples, including B cells, cancer-associated fibroblasts (CAFs), endothelial cells, macrophages, malignant epithelial cells, and T/NK cells. **(B)** Identification of malignant cells based on inferred copy number variation (CNV). The left panel shows the distribution of high-CNV (malignant) and low-CNV (normal-like) cells in UMAP space, while the right panel presents total CNV scores across different cell types or malignant epithelial subpopulations. **(C)** UMAP feature plots showing the expression patterns of the proliferation-related genes MKI67 and PLK1 at the single-cell level in CRC samples.

UMAP dimensionality reduction revealed multiple cell populations within the samples, among which malignant epithelial cells constituted the predominant fraction of the overall cell population (**Figure 4A**). Distinct separation of different cell types was observed in the low-dimensional space, indicating marked cellular heterogeneity within CRC tissues.CNV analysis showed that high-CNV cells were primarily enriched in the malignant epithelial cell population, whereas immune and stromal cells generally exhibited low-CNV profiles (**Figure 4B**). CNV scores also varied across malignant cell subpopulations, with the proliferative malignant subpopulation showing relatively higher CNV scores.Further analysis of gene expression patterns demonstrated that PLK1 was highly expressed in malignant epithelial cells, and its high-expression signals were mainly distributed in malignant cell clusters with elevated CNV levels (**Figure 4C**).

**Figure 5:**
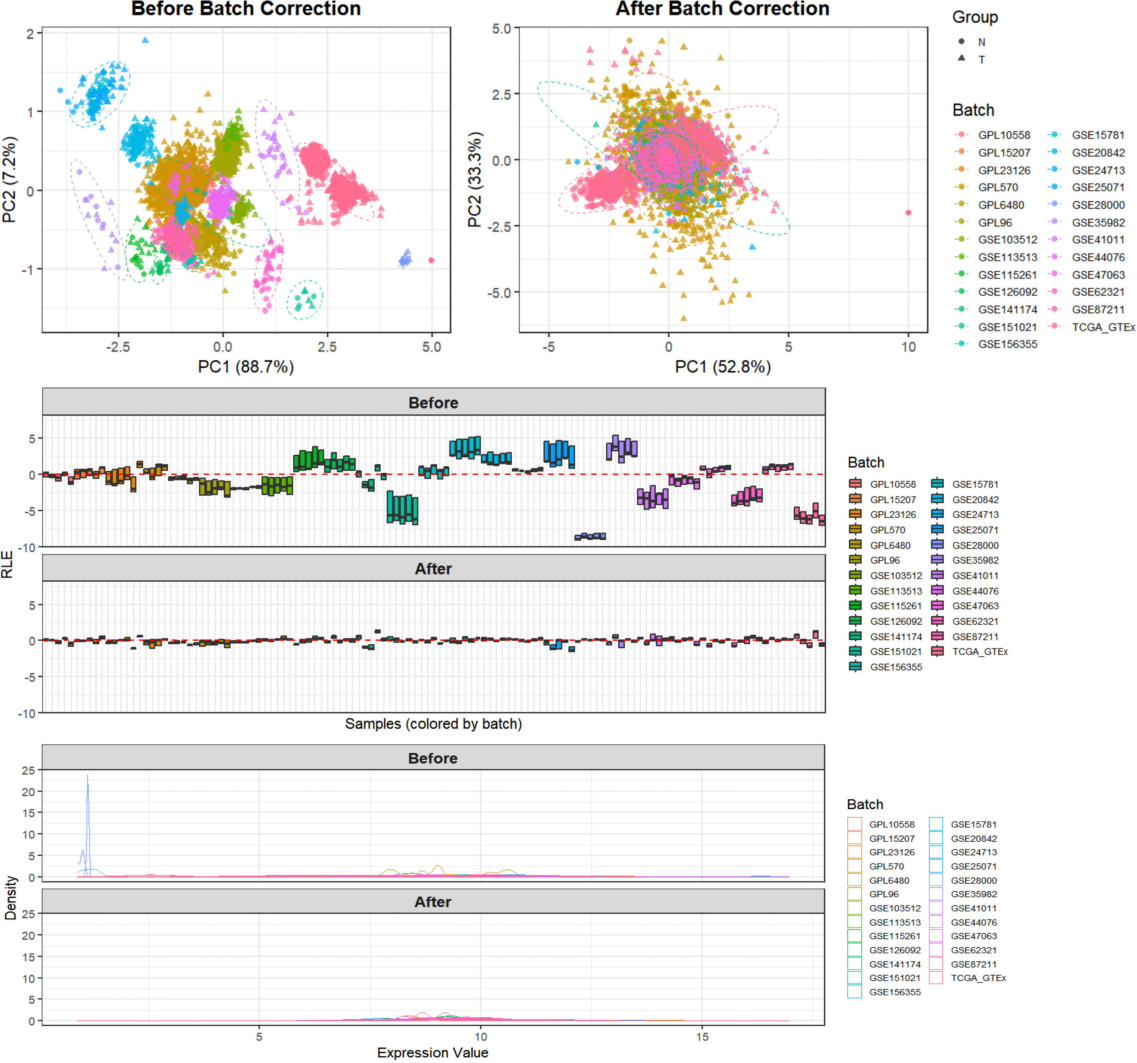
Batch effect assessment and correction in the integrated multi-platform colon cancer gene expression dataset for PLK1 biomarker analysis.

**Figure 6:**
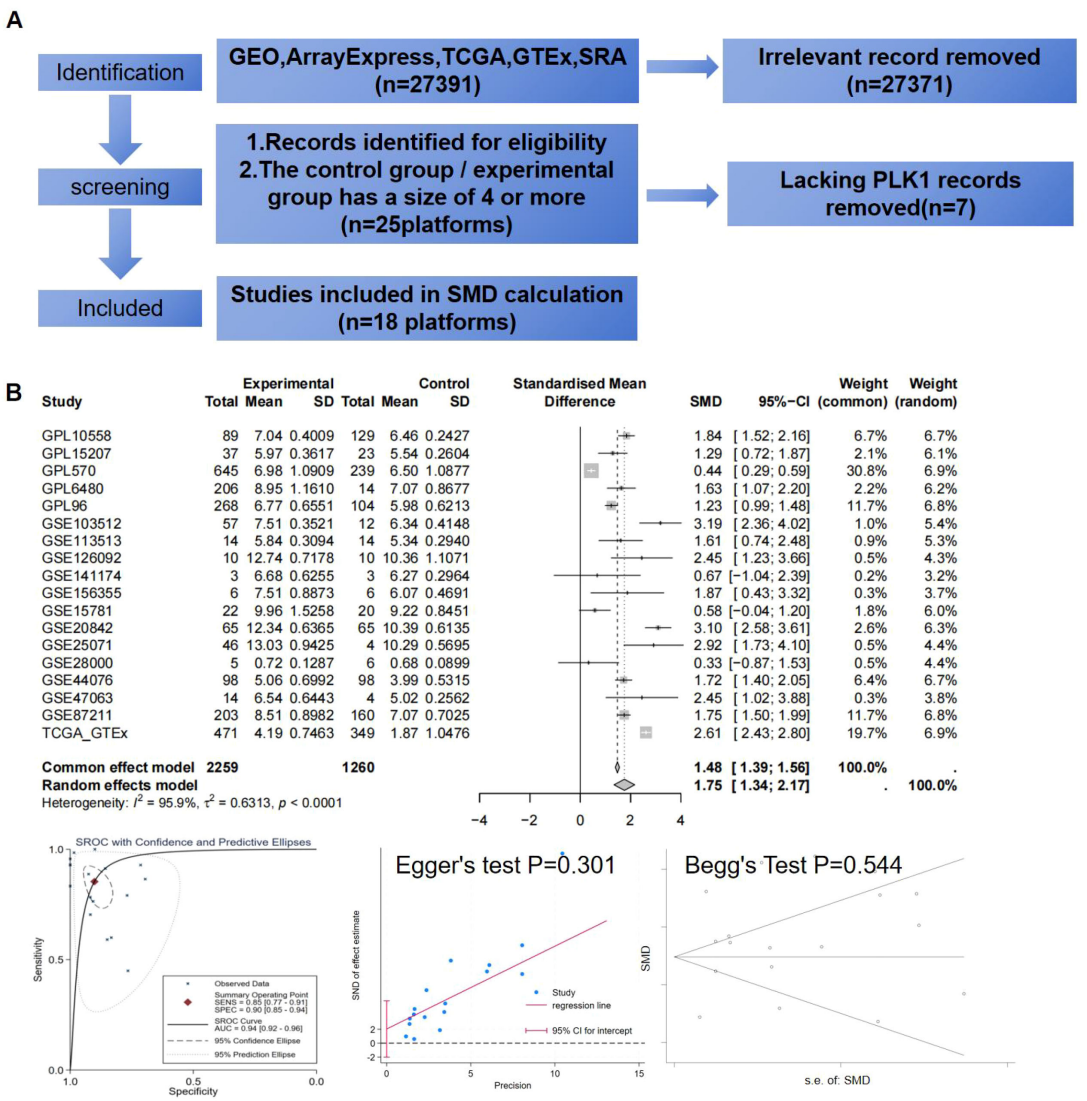
PLK1 is highly expressed at the mRNA level in CRC (non-CRC: 1260 samples; CRC: 2259 samples). **(A)** Inclusion flowchart of mRNA datasets. **(B)** SMD diagram, funnel plot, and SROC diagram. PLK1: polo-like kinase 1; CRC, colorectal cancer; SMD, standardized mean difference; SROC, summary receiver operating characteristic.

**Figure 7:**
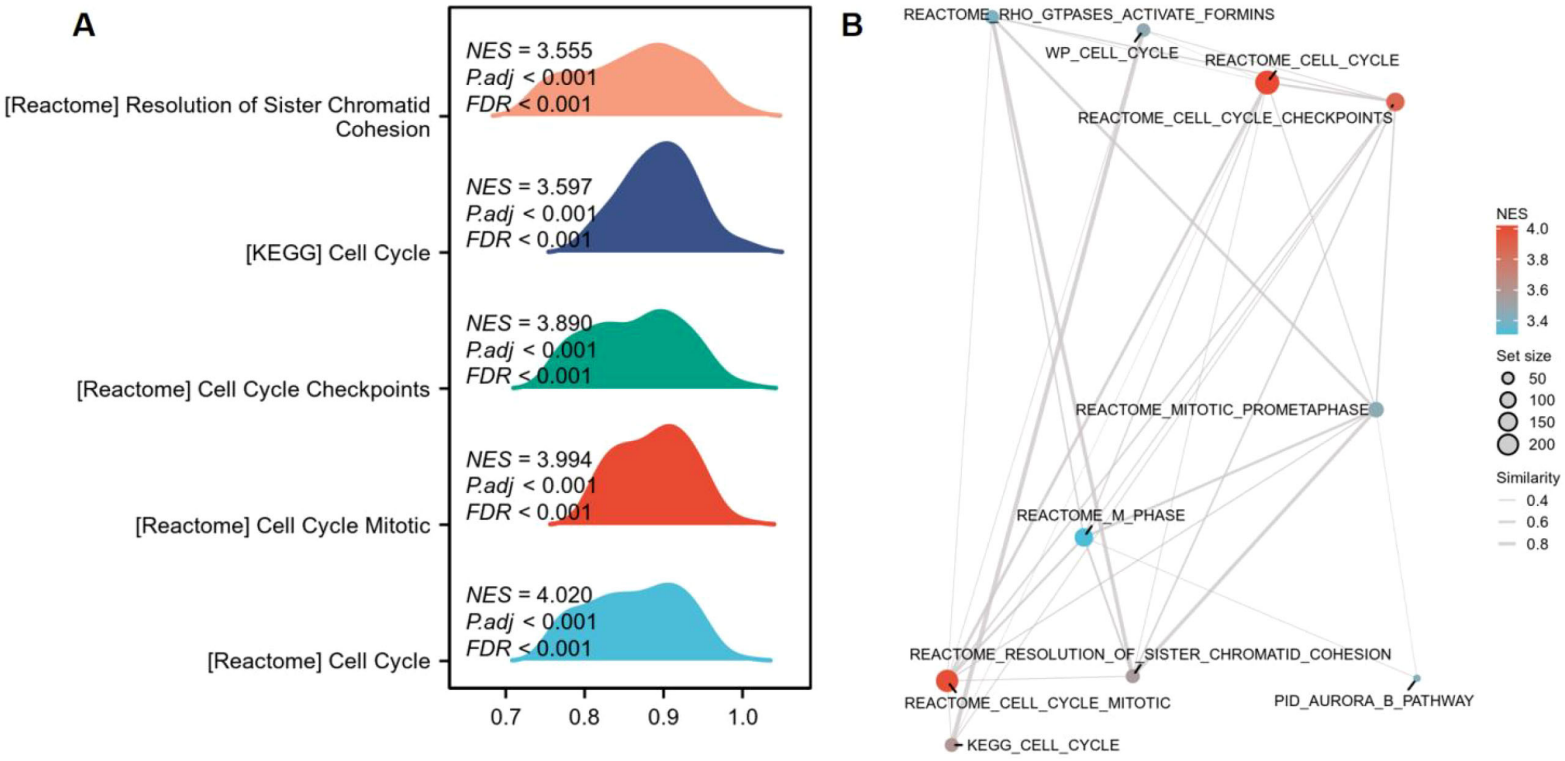
GSEA analysis of PLK1-coexpressed genes in CRC (R>0.5, P<0.05). **(A)** Pathway enrichment results. **(B)** Enrichment pathway network diagram. It shows the relationship between various pathways; the node size represents the number of gene sets, and the color represents the enrichment intensity, revealing that PLK1 is mainly involved in the cell cycle regulatory network. PLK1: polo-like kinase 1; CRC, colorectal cancer; GSEA, Gene Set Enrichment Analysis.

**Figure 8:**
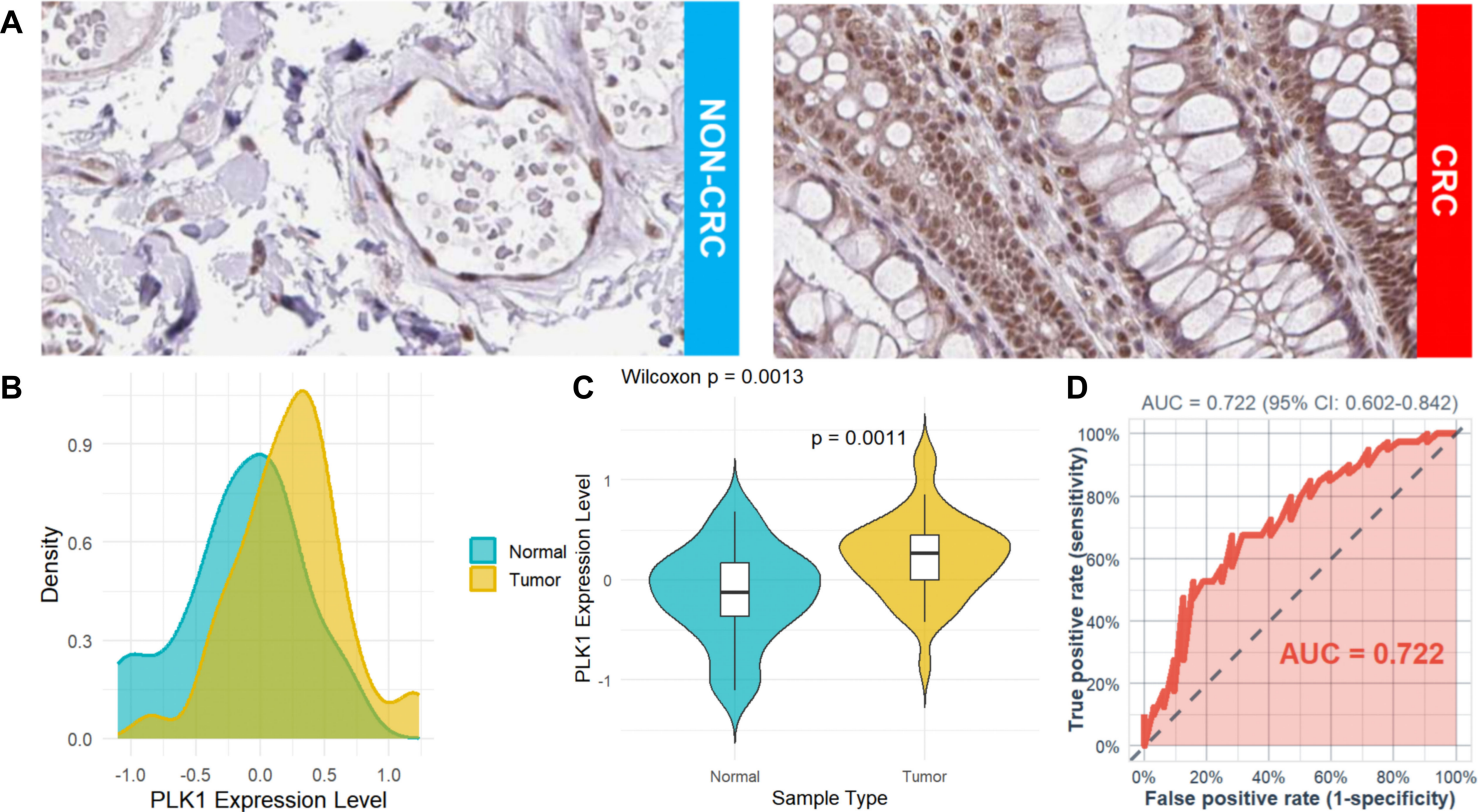
PLK1 is highly expressed at the protein level in CRC. **(A)** Immunohistochemical staining. **(B)** Expression density distribution diagram. **(C)** Comparison of expression levels. **(D)** ROC curve analysis. Data in B-D are from Proteomic Data Commons (30 normal cases vs 42 CRC cases). PLK1, polo-like kinase 1; CRC, colorectal cancer; ROC, receiver operating characteristic.

After integrating the multi-platform transcriptomic datasets, batch effects were evaluated and corrected. Before correction, samples from different platforms were clearly separated in the principal component analysis, and substantial deviations were observed in both RLE and overall expression distributions. After batch correction, the overlap among samples from different datasets increased in PCA space, the RLE values were centered more closely around 0, and the expression density distributions became more consistent, indicating that batch effects had been corrected (**Figure 5**). For the analysis of PLK1 expression differences, data from 18 platforms were included in the pooled SMD analysis, comprising 2,259 CRC samples and 1,260 non-CRC samples (**Figures 6A, B**). Using a pre-specified random-effects model, the pooled SMD was 1.75 (95% CI: 1.34–2.17), indicating higher PLK1 mRNA expression in CRC tissues compared with non-CRC tissues. Substantial between-study heterogeneity was observed (I² = 95.9%, τ² = 0.6313, P < 0.0001). The 95% prediction interval was wide and included values both below and above zero. SROC analysis yielded an AUC of 0.94 (95% CI: 0.92–0.96), with a sensitivity of 0.85 (0.77–0.91) and a specificity of 0.86 (0.85–0.94). The corresponding diagnostic contingency data (TP, FP, FN, and TN) for each included dataset are presented in **Table 1**. In addition, neither Egger’s test (P = 0.301) nor Begg’s test (P = 0.544) indicated significant publication bias (**Figure 6B**). PLK1-coexpressed genes (r > 0.5, P < 0.05) were enriched in cell cycle pathways (NES 3.5–4.0, all P < 0.001), forming a network centered on Cell Cycle and Cell Cycle Mitotic (**Figure 7**).Additionally, PLK1 protein was lowly expressed in non-CRC tissues and highly expressed in CRC tissues (**Figure 8A**). The expression level of PLK1 in tumor tissues was significantly higher than that in normal tissues: PLK1 mRNA expression in tumor tissues was notably higher than in normal tissues (Wilcoxon, P = 0.001), and the AUC for PLK1 expression level was 0.722 (95%CI: 0.602–0.842) (**Figures 8B–D**).

**Table 1:** Performance metrics of PLK1 in CRC (TP/FP/FN/TN values).

ID	TP	FP	FN	TN
GPL10558	68	12	21	117
GPL15207	32	7	5	16
GPL570	290	56	355	183
GPL6480	188	2	18	12
GPL96	212	24	56	80
GSE103512	53	0	4	12
GSE113513	13	4	1	10
GSE126092	10	1	0	9
GSE141174	3	2	0	1
GSE156355	5	0	1	6
GSE15781	13	3	9	17
GSE20842	64	1	1	64
GSE25071	44	0	2	4
GSE28000	3	1	2	5
GSE44076	69	8	29	90
GSE47063	13	0	1	4
GSE87211	159	13	44	147
TCGA_GTEx	418	26	53	323

**Figure 9:**
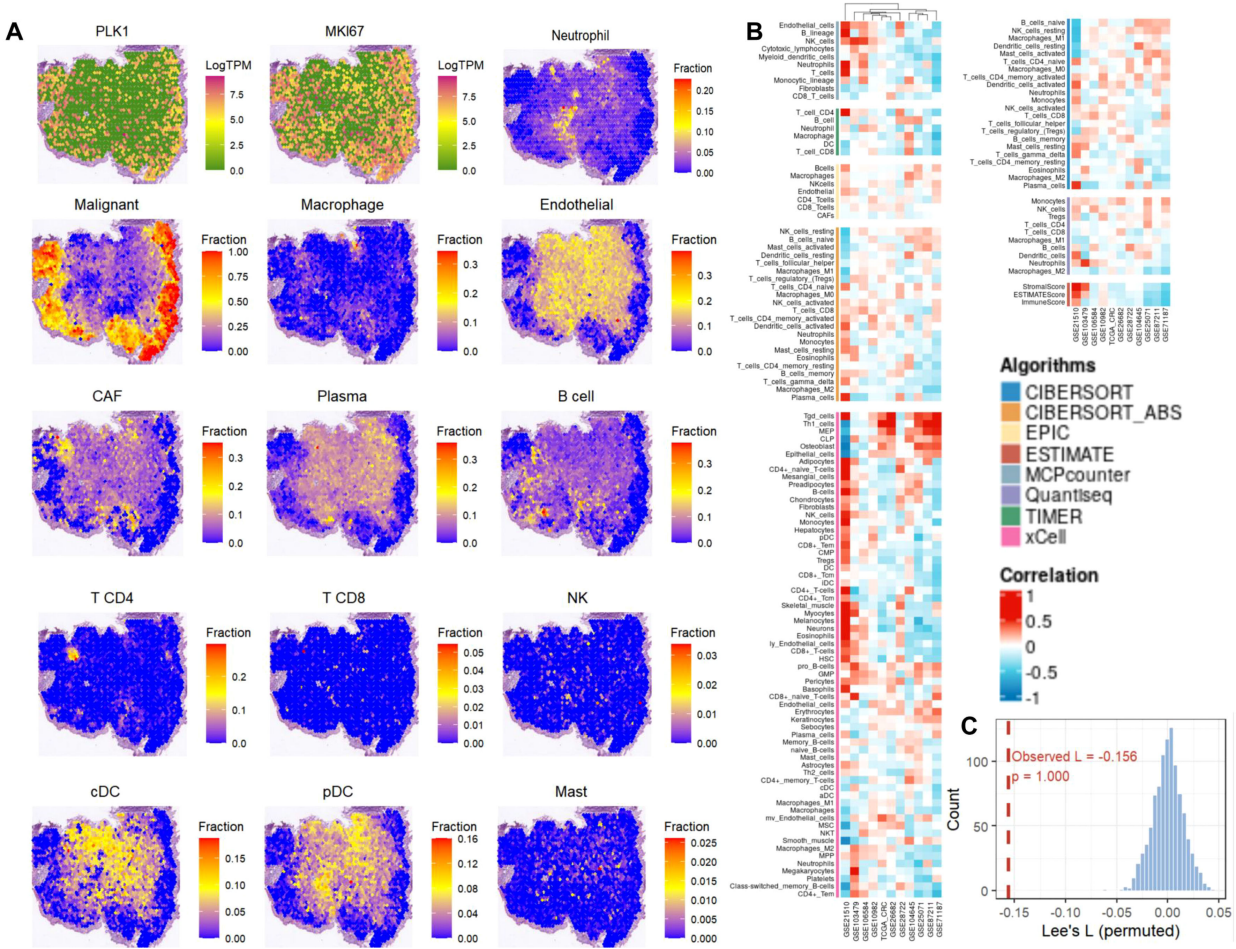
Spatial transcriptomics reveals PLK1-related spatial distribution patterns and microenvironmental features in tumor tissue. **(A)** Spatial maps showing LogTPM expression of proliferation markers PLK1 and MKI67, neutrophil fraction, and inferred cell-type fractions (malignant, macrophage, endothelial, CAF, plasma, B cell, CD4^+^ T cell, CD8^+^ T cell, NK cell, cDC, pDC, and mast cell). **(B)** Correlation heatmap of cell-type abundances estimated by multiple deconvolution algorithms (CIBERSORT, CIBERSORT_ABS, EPIC, ESTIMATE, MCPcounter, Quantiseq, TIMER, xCell) across GEO datasets. **(C)** Histogram of permuted Lee’s L spatial statistic (observed L = −0.156).

Spatial transcriptomic analysis showed that PLK1 and the proliferation marker MKI67 exhibited highly similar spatial distribution patterns across the tissue sections, with their high-expression regions mainly enriched in malignant cell-dominant areas (**Figure 9A**). Cell-type deconvolution further revealed that malignant cells were primarily localized within the tumor core regions, whereas endothelial cells, cancer-associated fibroblasts (CAFs), plasma cells, B cells, and some myeloid cell populations displayed heterogeneous spatial distributions. In contrast, CD4^+^ T cells, CD8^+^ T cells, NK cells, cDCs, pDCs, and mast cells showed relatively low overall abundance and were distributed in a more focal manner (**Figure 9A**).

**Figure 10:**
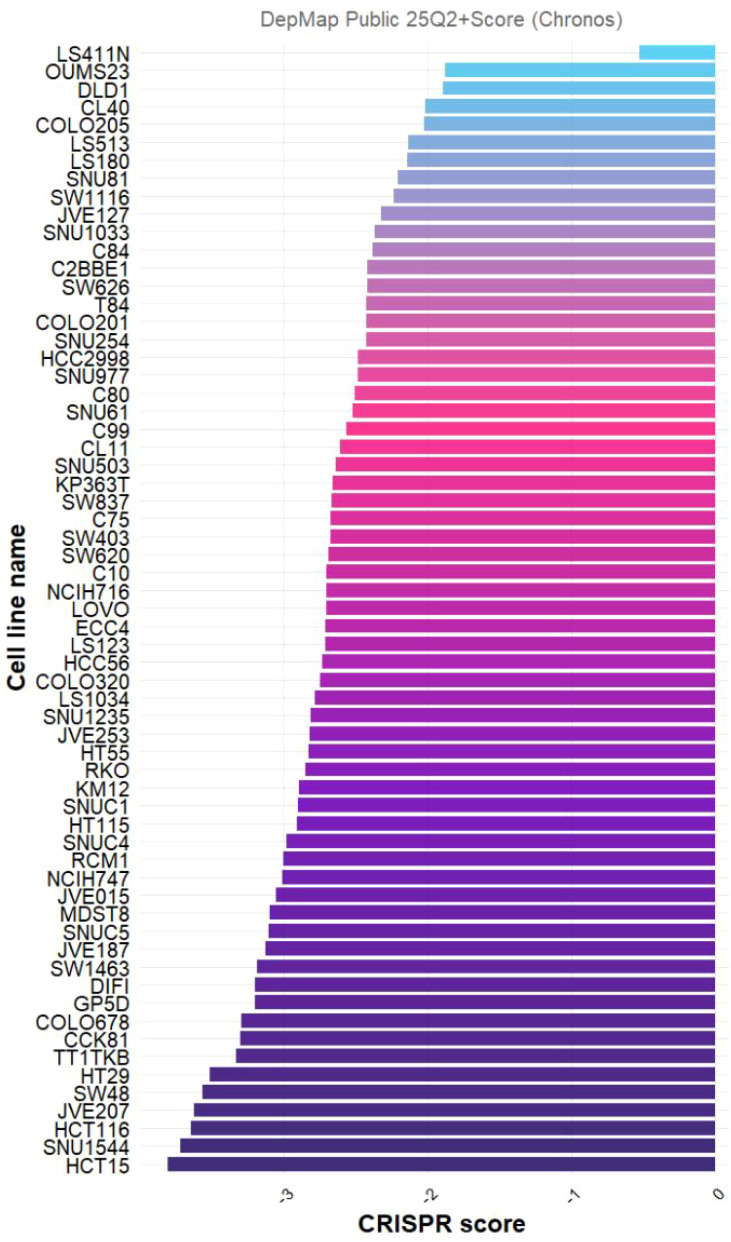
Knockdown of PLK1 inhibits CRC-related cells. A negative CRISPR score indicates that the gene is essential for cell survival, and a more negative score indicates a more significant inhibition of cell growth after knockdown. PLK1, polo-like kinase 1; CRC, colorectal cancer; CRISPR, clustered regularly interspaced short palindromic repeats.

To quantify spatial autocorrelation, univariate Moran’s I statistics were computed for PLK1 expression and for a composite immune score. PLK1 expression exhibited significant positive spatial autocorrelation (Moran’s I = 0.1632, p = 0.001), as did the composite immune score (Moran’s I = 0.5518, p = 0.001). After correcting for UMI count variation, the results remained significant (PLK1: Moran’s I = 0.1241, p = 0.001; immune score: Moran’s I = 0.4280, p = 0.001). Consistency analysis across eight deconvolution algorithms (CIBERSORT, CIBERSORT_ABS, EPIC, ESTIMATE, MCPcounter, quanTIseq, TIMER, and xCell) showed variable correlations between PLK1 expression and different immune/stromal populations (**Figure 9B**). Bivariate spatial autocorrelation between PLK1 expression and total immune infiltration was assessed using Lee’s L statistic. The observed Lee’s L was −0.1558 (raw data) and −0.0634 (UMI-corrected), both with a permutation test p = 1.000, indicating no statistically significant spatial co-localization or segregation between PLK1 expression and immune infiltration beyond what would be expected by chance (**Figure 9C**).CRISPR screening in DepMap revealed PLK1 dependency in CRC cell lines (most negative scores in HCT116, HCT15, SNU1544 ≈ -3.0), confirming essential role in cell growth (**Figure 10**).

### NC downregulates PLK1 expression

**Figure 11:**
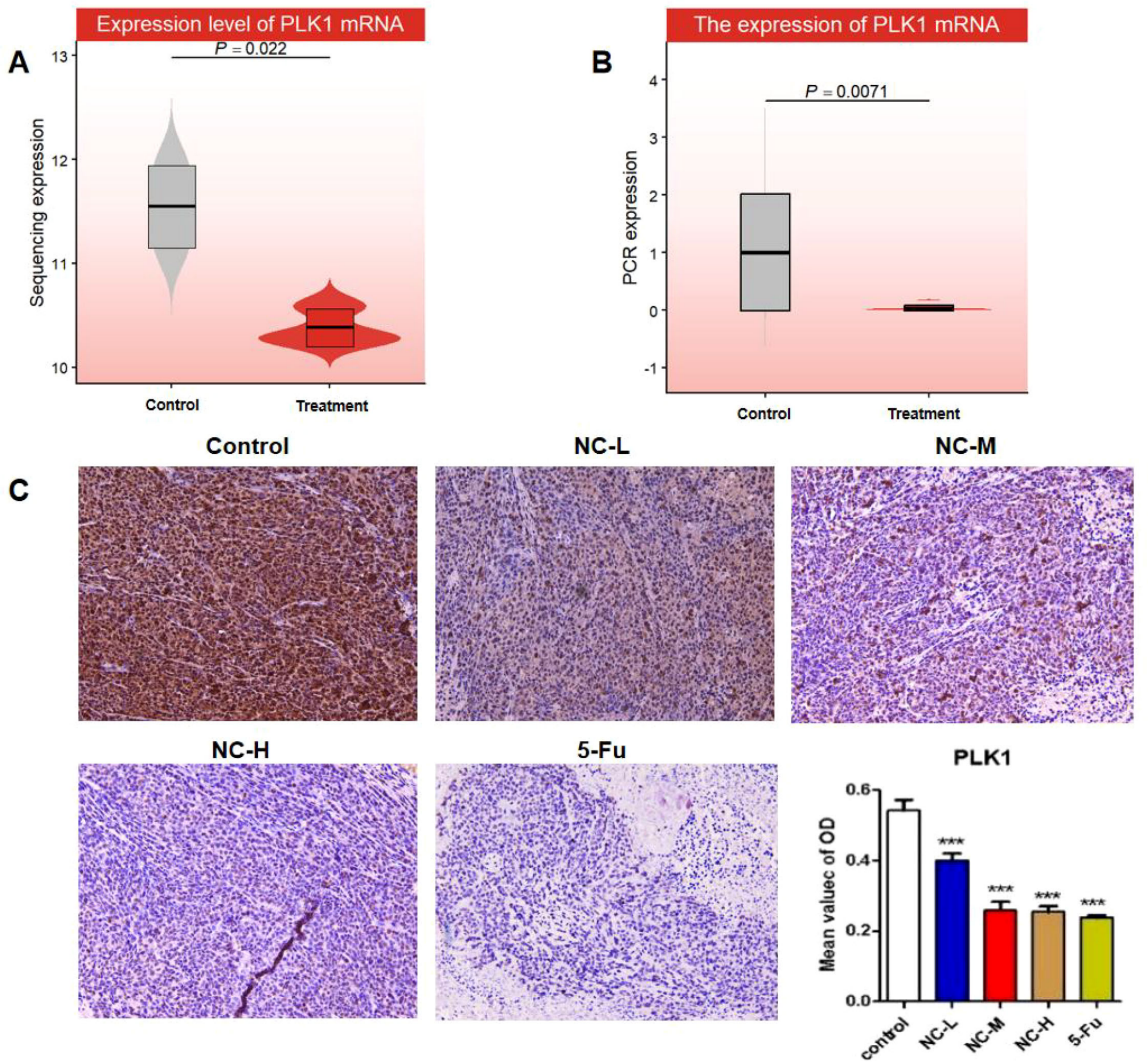
PLK1 is downregulated at the mRNA and protein levels after NC treatment. **(A)** Transcriptome sequencing verification. The mRNA expression of PLK1 in HCT116 cells was significantly downregulated after NC treatment. P < 0.01 vs control group. **(B)** RT-qPCR verification. The mRNA expression level of PLK1 was significantly reduced after treatment with NC (6 μmol/L) for 48h. Data are expressed as mean ± standard deviation. ***P < 0.001 vs control group. **(C)** *In vivo* immunohistochemical verification. Detection of PLK1 protein expression in ectopic xenograft tissues of mice. NC downregulated PLK1 protein expression in a dose-dependent manner, and the bar chart in the lower right corner shows the quantitative analysis of the average optical density value of each group. ***P < 0.001 vs control group. NC, Nitidine Chloride; PLK1, polo-like kinase 1.

RNA-seq showed PLK1 mRNA decreased in HCT116 cells after NC treatment (**Figure 11A**, P < 0.05). RT-qPCR confirmed significant reduction after 48 h 6 μM NC (**Figure 11B**, ***P < 0.001). *In vivo* IHC in HCT116 xenograft model demonstrated dose-dependent PLK1 protein downregulation by NC, with high-dose NC comparable to 5-FU (**Figure 11C**).

**Figure 12:**
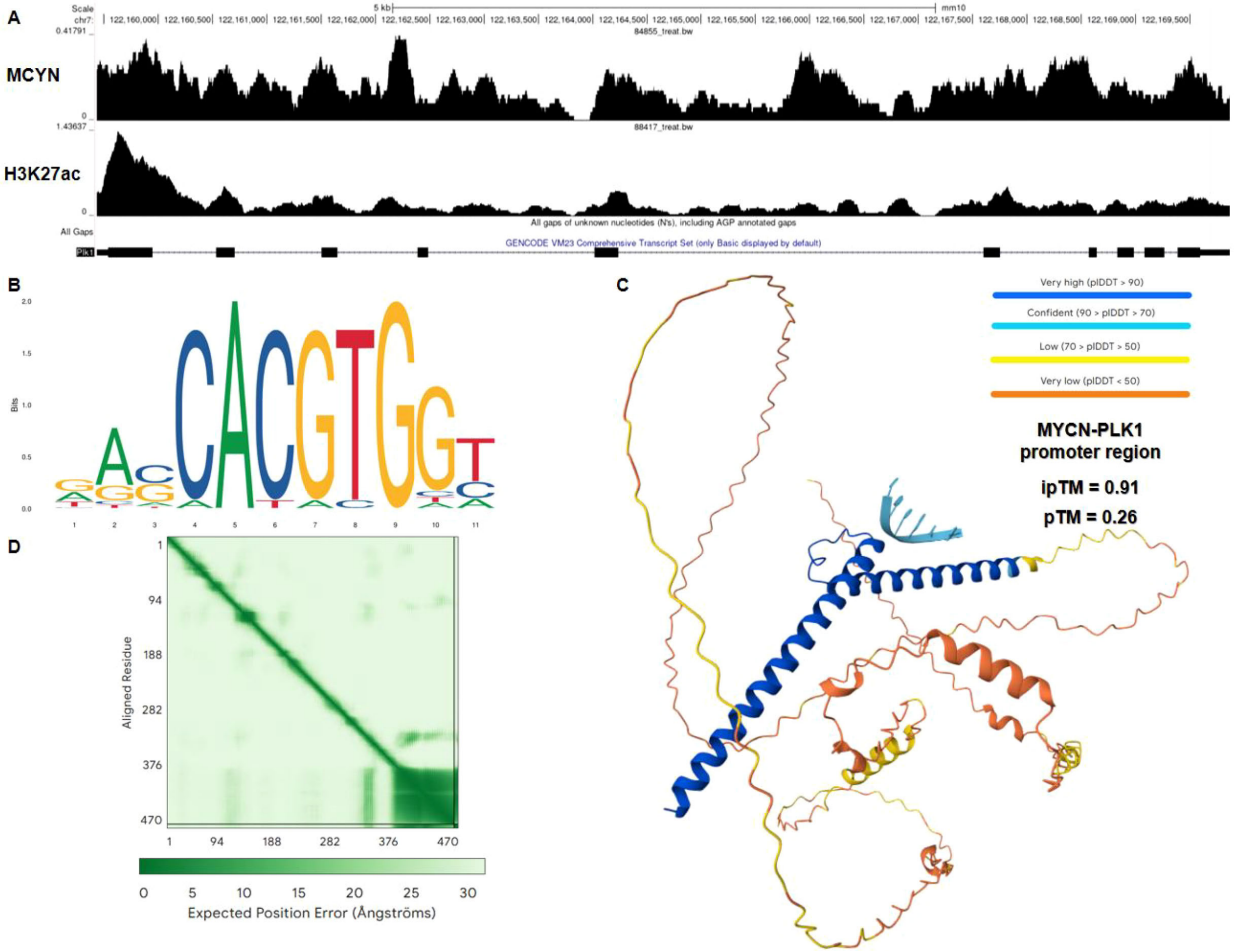
MYCN directly binds to the PLK1 promoter and both genes are downregulated by Nitidine Chloride. **(A)** Genome browser tracks showing MYCN ChIP-seq signal and H3K27ac enrichment across the PLK1 genomic locus. The genomic coordinates and gene annotation are shown below. **(B)** Sequence logo representing the predicted MYCN–MAX binding motif (E-box) identified within the PLK1 promoter region. **(C)** Structural model of the MYCN–MAX complex in association with the PLK1 promoter DNA sequence generated by AlphaFold-Multimer and protein–DNA docking. The structure is colored according to pLDDT confidence scores. **(D)** Predicted aligned error (PAE) plot of the MYCN–MAX complex, indicating the confidence of relative positioning between residues.

Given that both MYCN and PLK1 were significantly downregulated following Nitidine Chloride treatment, we next investigated their potential regulatory relationship. Transcriptomic sequencing showed that MYCN was significantly downregulated after Nitidine Chloride treatment, with logFC = -1.5579, logCPM = -1.2206, and P = 0.0057. Together with the previously observed downregulation of PLK1, these findings indicated a concurrent decrease in MYCN and PLK1 expression after Nitidine Chloride treatment. Genome browser visualization showed MYCN occupancy near the PLK1 locus, and the MYCN ChIP-seq peaks overlapped with regions marked by H3K27ac, indicating active chromatin features around the PLK1 promoter region (**Figure 12A**). Motif analysis further identified a canonical E-box sequence within the PLK1 promoter, consistent with the known DNA-binding preference of the MYCN–MAX complex (**Figure 12B**). Structural modeling showed that the MYCN–MAX heterodimer adopted a stable bHLH conformation (**Figure 12C**). In addition, protein–DNA complex prediction showed a visualizable interaction configuration between the MYCN–MAX complex and the PLK1 promoter region, with the relevant model parameters shown in **Figure 12D**.

**Figure 13:**
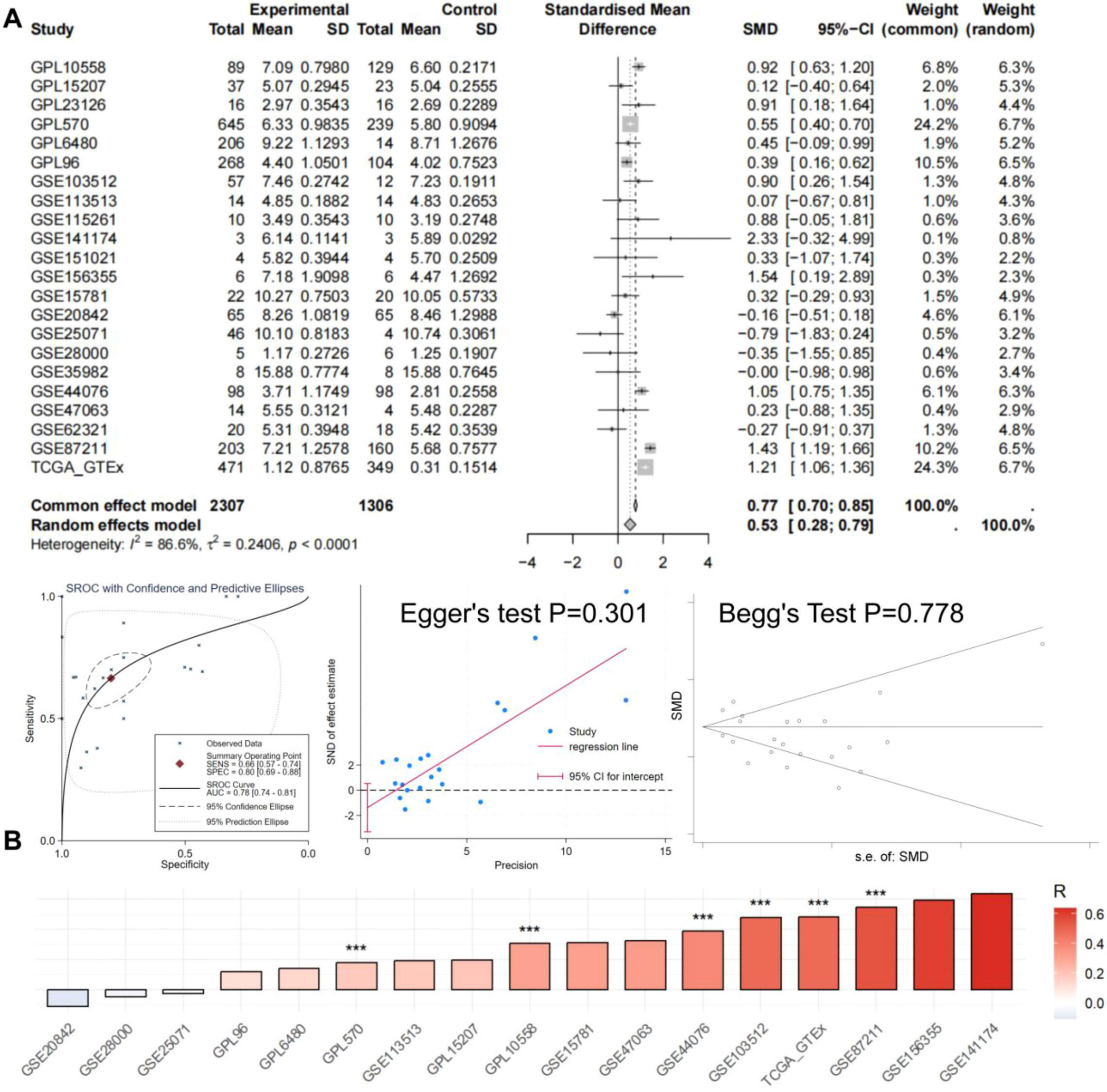
High MYCN mRNA expression in CRC. **(A)** Forest plot showing the standardized mean difference (SMD) in MYCN mRNA expression levels between CRC (experimental) and normal control groups across multiple studies/datasets, with pooled estimates for common-effect and random-effects models, heterogeneity statistics, summary receiver operating characteristic (SROC) curve, and publication bias tests (Egger’s and Begg’s). **(B)** Heatmap of Pearson correlation coefficients (R) between PLK1 and MYCN mRNA expression across the listed studies (significant correlations marked with asterisks).

We further performed an integrated multi-cohort analysis of MYCN expression in CRC. A total of 22 platforms were included in the pooled SMD analysis, comprising 2,307 CRC samples and 1,306 normal control samples (**Figure 13A**). The random-effects pooled SMD was 0.53 (95% CI: 0.28–0.79), indicating a modest overall trend toward higher MYCN mRNA expression in CRC tissues compared with normal tissues. However, substantial between-study heterogeneity was observed (I² = 86.6%, τ² = 0.2406, P < 0.0001), and 9 of the 22 studies had 95% confidence intervals that crossed zero. The prediction interval for the pooled estimate was wide and included zero, suggesting that a future study could plausibly show either an increase or no difference in MYCN expression. SROC analysis yielded an AUC of 0.78 (95% CI: 0.74–0.81), with a sensitivity of 0.68 (0.57–0.74) and a specificity of 0.80 (0.69–0.86). The detailed TP, FP, FN, and TN values for each included dataset are summarized in **Table 2**. In addition, neither Egger’s test (P = 0.301) nor Begg’s test (P = 0.778) indicated significant publication bias (**Figure 13A**). Pearson correlation analysis was performed between PLK1 and MYCN mRNA expression across 14 independent CRC cohorts (**Table 3**; **Figure 13B**). Positive correlations were observed in 12 of the 14 cohorts, with correlation coefficients ranging from 0.116 to 0.634. After Benjamini-Hochberg FDR correction for multiple testing, statistically significant positive correlations (FDR < 0.05) were retained in 6 cohorts, including the strongest associations in GSE87211 (R = 0.544), TCGA_GTEx (R = 0.481), and GSE103512 (R = 0.479). The remaining cohorts showed weak or non-significant correlations. These results indicate a consistent moderate positive association between MYCN and PLK1 expression in the majority of CRC cohorts.

**Table 2:** Performance metrics of MCYN in CRC (TP/FP/FN/TN values).

ID	TP	FP	FN	TN
GPL10558	52	11	37	118
GPL15207	26	12	11	11
GPL23126	8	0	8	16
GPL570	458	119	187	120
GPL6480	78	2	128	12
GPL96	80	8	188	96
GSE103512	38	2	19	10
GSE113513	14	10	0	4
GSE115261	7	2	3	8
GSE141174	3	0	0	3
GSE151021	3	1	1	3
GSE156355	5	0	1	6
GSE15781	8	2	14	18
GSE20842	45	37	20	28
GSE25071	41	1	5	3
GSE28000	5	4	0	2
GSE35982	4	2	4	6
GSE44076	61	13	37	85
GSE47063	8	1	6	3
GSE62321	16	10	4	8
GSE87211	136	9	67	151
TCGA_GTEx	315	16	156	333

**Table 3:** Pearson correlation between PLK1 and MYCN mRNA expression across 14 independent CRC cohorts.

Cohort	R	p.value	FDR
GSE141174	0.634	0.1760993	0.2340075
GSE156355	0.593	0.04205369	0.07632702
GSE87211	0.544	2.61784E-29	2.22517E-28
TCGA_GTEx	0.481	1.05272E-48	1.78962E-47
GSE103512	0.479	3.08399E-05	8.73796E-05
GSE44076	0.387	2.10083E-08	1.19047E-07
GSE47063	0.324	0.1893323	0.2340075
GSE15781	0.311	0.04489825	0.07632702
GPL10558	0.305	4.61578E-06	1.56937E-05
GPL15207	0.196	0.1336605	0.2065663
GSE113513	0.189	0.3365552	0.3814293
GPL570	0.18	6.83012E-08	2.9028E-07
GPL6480	0.139	0.03873632	0.07632702
GPL96	0.116	0.02530874	0.06146408
GSE25071	-0.028	0.8494591	0.8836653
GSE28000	-0.05	0.8836653	0.8836653
GSE20842	-0.115	0.192712	0.2340075

## Discussion

Transcriptomic analysis revealed that cell cycle-related genes were significantly downregulated after NC treatment, among which PLK1 was identified as a key regulatory factor and an important target of NC. MDS confirmed the stable binding between NC and PLK1, which forms a complex mainly through van der Waals interactions. Multi-omics analysis revealed the high expression characteristics of PLK1 in CRC from the single-cell level to the tissue level.Integrated analyses further showed that PLK1 was predominantly enriched in malignant epithelial cells and highly proliferative tumor regions, and its expression pattern was spatially consistent with that of MKI67. In addition, public dependency screening data indicated that CRC cell lines exhibited marked dependence on PLK1, further supporting its relevance in CRC cell growth.

The direct inhibitory effect of NC on PLK1 triggers a cascade of downstream molecular events. As a key regulator of the G2/M phase transition of the cell cycle, the inhibition of PLK1 activity directly affects the normal progression of cell division (22, 23). Under normal circumstances, PLK1 activates the CDK1-Cyclin B complex by phosphorylating the CDC25C phosphatase, thereby promoting the cell cycle transition from the G2 phase to the M phase. When NC binds to and inhibits PLK1, this phosphorylation event is blocked, resulting in insufficient activation of the CDK1-Cyclin B complex and subsequent cell cycle arrest at the G2/M checkpoint (24). Meanwhile, the inhibition of PLK1 also impairs the activation of the Anaphase-Promoting Complex/Cyclosome (APC/C) (25); since APC/C is responsible for degrading cyclins and securin inhibitors, its dysfunction further exacerbates cell cycle arrest. In addition, PLK1 plays a crucial role in centrosome maturation and spindle formation; thus, its inhibition leads to the activation of the spindle checkpoint, triggering the apoptosis program or causing catastrophic cell death during mitosis. The anti-tumor effect of NC also involves the interactive regulation of multiple signal transduction pathways. The p53-p21 pathway (26), a core mechanism for cell cycle control, may be activated under the action of NC: DNA damage and replication stress caused by PLK1 inhibition activate ATM/ATR kinases (27), which in turn phosphorylate and activate p53. As a transcription factor, activated p53 upregulates the expression of cell cycle inhibitors such as p21 to further enhance G2/M phase arrest; simultaneously, it activates the transcription of pro-apoptotic genes (e.g., BAX, PUMA, and NOXA) and initiates the mitochondria-dependent apoptotic pathway. Furthermore, the inhibition of PLK1 may affect the PI3K/AKT signaling pathway (28); PLK1 negatively regulates the phosphatase activity of PTEN through phosphorylation (29); when PLK1 is inhibited, PTEN activity is enhanced, leading to the downregulation of the AKT signaling pathway and ultimately affecting the activity of cell survival-related proteins such as MDM2 and GSK3β (30).

From the perspective of clinical translation, the anti-tumor effect of NC by regulating the PLK1 axis holds important application prospects. Currently, a variety of PLK1 inhibitors have entered the clinical trial stage, but the main challenges of existing drugs include insufficient specificity and dose-limiting toxicity, especially the adverse effects on the hematopoietic system and gastrointestinal tract (7). As a natural compound, NC has the characteristic of multi-target action, which may provide a wider therapeutic window. The regulation of PLK1 by NC may not only enhance the inhibitory effect on PLK1 but also increase the sensitivity of chemotherapy drugs by affecting DNA repair mechanisms, offering new opportunities for combination therapy strategies. However, to realize the clinical translation of NC, further studies on its pharmacokinetic characteristics (including bioavailability, metabolic pathways, and toxicity profile) are required. Meanwhile, the development of biomarkers based on PLK1 expression levels will help identify patient populations most likely to benefit from NC treatment, thereby achieving the goal of precision medicine.

In addition to its cell cycle–related functions, emerging evidence from our integrative analyses suggests that PLK1 may play a broader role within the spatial and regulatory architecture of CRC. Spatial transcriptomic profiling revealed that PLK1 expression is not uniformly distributed across tumor tissues but instead concentrates in highly proliferative tumor niches and overlaps with malignant cell-enriched regions. Univariate spatial autocorrelation analysis confirmed significant positive spatial clustering for both PLK1 expression and the composite immune score, indicating that PLK1-high areas and immune-infiltrated areas each tend to form independent spatial clusters. Consistency analysis across multiple deconvolution algorithms further demonstrated variable correlations between PLK1 and different immune/stromal components. Bivariate spatial autocorrelation analysis showed no significant spatial association between PLK1 expression and overall immune infiltration, suggesting that while both features are spatially clustered, they do not significantly co-localize or segregate at the tissue scale. This result is consistent with the deconvolution-based correlation patterns, which showed relatively consistent positive correlations with certain myeloid and endothelial populations but weaker correlations with lymphoid subsets. Collectively, these findings indicate that PLK1-driven proliferation is embedded within specific tumor microenvironmental contexts, linking cell cycle regulation with spatially heterogeneous microenvironmental features without requiring direct spatial overlap with total immune infiltration. Furthermore, in silico analyses suggest a potential transcriptional association between MYCN and PLK1. Transcriptomic sequencing showed that MYCN was significantly downregulated after NC treatment, occurring in parallel with the reduction of PLK1. Integrated meta-analysis across multiple independent CRC cohorts revealed a modest overall trend toward higher MYCN expression in tumor tissues. Genome browser visualization, motif analysis, and structural modeling further supported a plausible interaction between the MYCN–MAX complex and the PLK1 promoter region. Taken together, these findings suggest that the MYCN–PLK1 axis may be involved in the inhibitory effects of NC on CRC.

## Limitations

Several limitations of this study should be acknowledged. First, although molecular docking, molecular dynamics simulation, genome browser visualization, motif analysis, and structural modeling supported the association of NC with PLK1 and suggested a potential MYCN–PLK1 regulatory relationship, these findings were primarily based on computational and integrative bioinformatics analyses, and additional experimental validation is still required. In addition, although the proposed MYCN–PLK1 transcriptional axis is novel and is supported by public ChIP-seq data, motif analysis, and AlphaFold-Multimer-based structural modeling, it has not yet been functionally validated in the present study. Further experiments, including luciferase reporter assays, ChIP-qPCR, and evaluation of PLK1 expression following MYCN overexpression or knockdown, will be necessary to confirm the direct transcriptional regulation of PLK1 by MYCN. Second, although PLK1 downregulation was validated by RNA-seq, RT-qPCR, and *in vivo* IHC after NC treatment, the direct transcriptional regulation of PLK1 by MYCN has not yet been confirmed by dedicated assays. Third, the public multi-omics datasets included in this study were derived from different platforms and cohorts, and although batch correction and integrative analyses were performed, underlying heterogeneity among datasets may still have affected the pooled estimates. Fourth, the spatial transcriptomic and deconvolution analyses mainly provided descriptive evidence regarding the distribution of PLK1-related proliferative niches and microenvironmental components, whereas the functional contribution of these spatial features remains to be further clarified. Finally, the pharmacokinetic properties, systemic toxicity, and clinical applicability of NC require further investigation before translation into clinical practice.

## Conclusion

In this study, we systematically investigated the potential anti-CRC mechanism of NC through transcriptomic sequencing, integrated multi-omics analyses, single-cell and spatial transcriptomics, molecular docking and molecular dynamics simulations, as well as *in vitro* and *in vivo* validation. The results identified PLK1 as an important candidate target of NC. Genes downregulated after NC treatment were significantly enriched in cell cycle-related processes, and PLK1 was consistently positioned as a key gene across multiple cell cycle-associated pathways. Molecular simulation analyses further showed a stable interaction pattern between NC and PLK1. Integrated multi-platform analyses demonstrated that PLK1 was highly expressed at both the mRNA and protein levels in CRC tissues, and single-cell and spatial transcriptomic analyses showed that PLK1 expression was mainly enriched in malignant epithelial cells and proliferative tumor regions. Deconvolution and spatial analyses further characterized the distribution of PLK1-associated microenvironmental components within tumor tissues. In addition, DepMap CRISPR screening revealed a marked dependency of CRC cell lines on PLK1. Consistently, RNA-seq, RT-qPCR, and xenograft IHC results showed that NC reduced PLK1 expression at both the transcriptional and protein levels. Further analyses showed that MYCN was also downregulated after NC treatment, and MYCN expression was positively correlated with PLK1 expression across public CRC cohorts, suggesting that the MYCN–PLK1 axis may be involved in the inhibitory effects of NC on CRC. Collectively, these findings indicate that NC may exert anti-CRC activity by suppressing PLK1 and its associated cell cycle regulatory network, with the MYCN–PLK1 axis representing a potential mechanistic component of this process.

## Data Availability

The datasets presented in this study can be found in online repositories. The names of the repository/repositories and accession number(s) can be found below: https://www.ncbi.nlm.nih.gov/, PRJNA1405062.
